# The landscape of ADAMTS13-related tests in thrombotic thrombocytopenic purpura: a review

**DOI:** 10.3389/fmed.2026.1829389

**Published:** 2026-04-30

**Authors:** Chan Meng, Konstantine Halkidis

**Affiliations:** Division of Hematologic Malignancies and Cellular Therapeutics, Department of Internal Medicine, Institute for Reproductive and Developmental Sciences, University of Kansas Medical Center, Kansas City, KS, United States

**Keywords:** ADAMTS 13, antibodies, autoimmune disease, thrombotic microangiopathy (TMA), TTP (thrombotic thrombocytopenic purpura)

## Abstract

Thrombotic thrombocytopenic purpura (TTP), an insidious and often devastating thrombotic microangiopathy, represents one of the few true hematologic emergencies. Since the identification of the von Willebrand factor (VWF) cleaving protease ADAMTS13 in the early 21st century, tremendous advances have been made in the diagnosis and treatment of the disease. However, as with many areas of science, new knowledge begets new questions about the pathophysiology of both congenital and immune TTP, which identify gaps in our understanding of the mechanistic effects of ADAMTS13 and its inhibitors. Improvements in the clinical management of patients that have—or are suspected to have—TTP requires further advances in diagnostic testing and identification of clinically relevant biomarkers. In this review, we discuss ADAMTS13-related diagnostic testing in TTP, identify the underlying assumptions that guide the field, and explore avenues of research that promise to improve the lives of patients with the disease.

## 1 Introduction

Thrombotic thrombocytopenic purpura (TTP) is a life-threatening disorder, first described by Moschcowitz in 1924 (1). Its pathology is characterized by catastrophic intravascular hemolysis, microangiopathic thrombosis, and disturbance in coagulation; patients often manifest fever, renal insufficiency, central nerve involvement, thrombocytopenia, hemolytic anemia, thrombosis and multiple organ failure (2–5). As a thrombotic microangiopathy (TMA), laboratory findings are characterized by schistocytes on peripheral blood smear representative of microangiopathic hemolytic anemia; severe thrombocytopenia; and increases in levels of lactate dehydrogenase (LDH), direct bilirubin, and haptoglobin (4–7).

The discovery and isolation of the metalloprotease ADAMTS13 (a disintegrin and metalloproteinase with thrombospondin motif 13) by different teams at the turn of century unveiled the prime culprit of the fatal disease (8). The ADAMTS13 gene is located on chromosome 9q34 (8), and ADAMTS13 is synthesized within hepatic stellate cell and in endothelial cells (9–11). ADAMTS13 is responsible for the cleavage of von Willebrand factor (VWF) multimers at Y1605-M1606 in the A2 domain, which mitigates VWF’s role in adherence, aggregation, and activation of platelets. Deficiency or dysfunction of ADAMTS13 causes ultra large VWF multimer clumping, blockage of micro-vessels, and subsequent symptoms (12–14).

The incidence of TTP is 1–6 per million each year; although TTP is a rare disease, the prevalence is likely underestimated (4, 15–18). About 95% of TTP cases are immune TTP (iTTP), which is caused by auto-immune antibodies (iTTP) targeting ADAMTS13; less than 5% of TTP cases are due to homozygous mutations in the ADAMTS13 allele, which manifests as congenital TTP (cTTP) (19, 20). In addition, a third category, of which the mechanism remains unknown and is thought to be mostly secondary to other underlying diseases, has also been described as unidentified TTP (uTTP) (15, 21), though iTTP and cTTP are far more common.

For iTTP, triple therapy of therapeutic plasma exchange (TPE), immunosuppression (steroids, anti-CD20, and cytotoxic reagents), and caplacizumab improved mortality from acute phase disease from well over 50% and as high as 90% down to about 10–20% (22–28). To this day, recognition of the possibility of TTP and the start of early management remain essential life-saving factors (20). However, complicating matters, the symptoms and course of disease of TTP often overlap with other thrombotic microangiopathies, such as hemolytic uremic syndrome (HUS), disseminated intravascular coagulation (DIC), hemolysis-elevated liver enzymes-low platelet (HELLP) syndrome, and catastrophic antiphospholipid syndrome (CAPS). It is important to differentiate the conditions not only for initiating the proper treatment, but also preventing unnecessary interventions such as TPE which are associated with their own risk of morbidity in patients who often have extremely severe thrombocytopenia (20, 29, 30). Among iTTP patients, about 20–50% experience at least one relapse (31–33).

In early years, the diagnosis of TTP relied on symptoms and clinical decision, yet the diversity of clinical manifestations spectrum posed a challenge to clinicians (4, 15, 16, 34, 35). The classical pentad of fever, hemolytic anemia, thrombocytopenia, renal failure, and neurologic dysfunction were found in less than 5% of patients at presentation (5). Based on laboratory parameters, scoring systems were developed. The Bentley score was the first, but because of its complexity and lack of external validation, it is not widely used (30, 36). In the modern day, the French Score and PLASMIC Score are commonly employed in clinical assessment, using non-ADAMTS13 related variables including medical history (i.e., history of cancer or organ transplant), and laboratory variables (i.e., platelet count, serum creatinine) (37–39). As we discuss below, ADAMTS13 activity assays have revolutionized the care of these patients, but such testing is still not universally available at all medical centers and can take as long as a week for ADAMTS13 results to be available when external laboratories are utilized for testing (40). The International Society of Thrombosis and Hemostasis (ISTH) recommends that because of the high potential for death in patients with TTP who remain untreated, for patients with a clinical picture of TMA and high suspicion of TTP in whom rapid ADAMTS13 testing is not available, clinicians should consider emergent initiation of TPE and corticosteroids; as such, the French and PLASMIC scores continue to have utility in aiding clinicians’ judgment in such scenarios (27, 29). Despite this, the majority of patients who are empirically treated for TTP do not have the disease, exposing them to risks of TPE including bleeding, infection, and anaphylaxis (41). We highlight this in part to discuss the central role of ADAMTS13 in TTP diagnosis and management and the clear importance of having rapidly available ADAMTS13 activity results.

In this review, we explore the landscape of ADAMTS13-related diagnostic testing available to guide the management of patients suspected of having TTP, as well as laboratory techniques used in current research to enhance our understanding of the pathophysiology of the disease. We describe ADAMTS13 activity assays and how they have revolutionized the care of TTP patients; assays to differentiate cTTP from iTTP; state-of-the-art knowledge about the role of different immunoglobin classes and subclasses in the pathophysiology of the immune form of the disease; and the vanguard of research approaches that may emerge into the next wave of clinically utilized innovations. Though this is not meant to be an all-encompassing review, we endeavor to highlight the breadth and depth of the accomplishments of the many people who have helped usher in the current era and whose work will define the future landscape of TTP care.

## 2 ADAMTS13 activity

**FIGURE 1:**
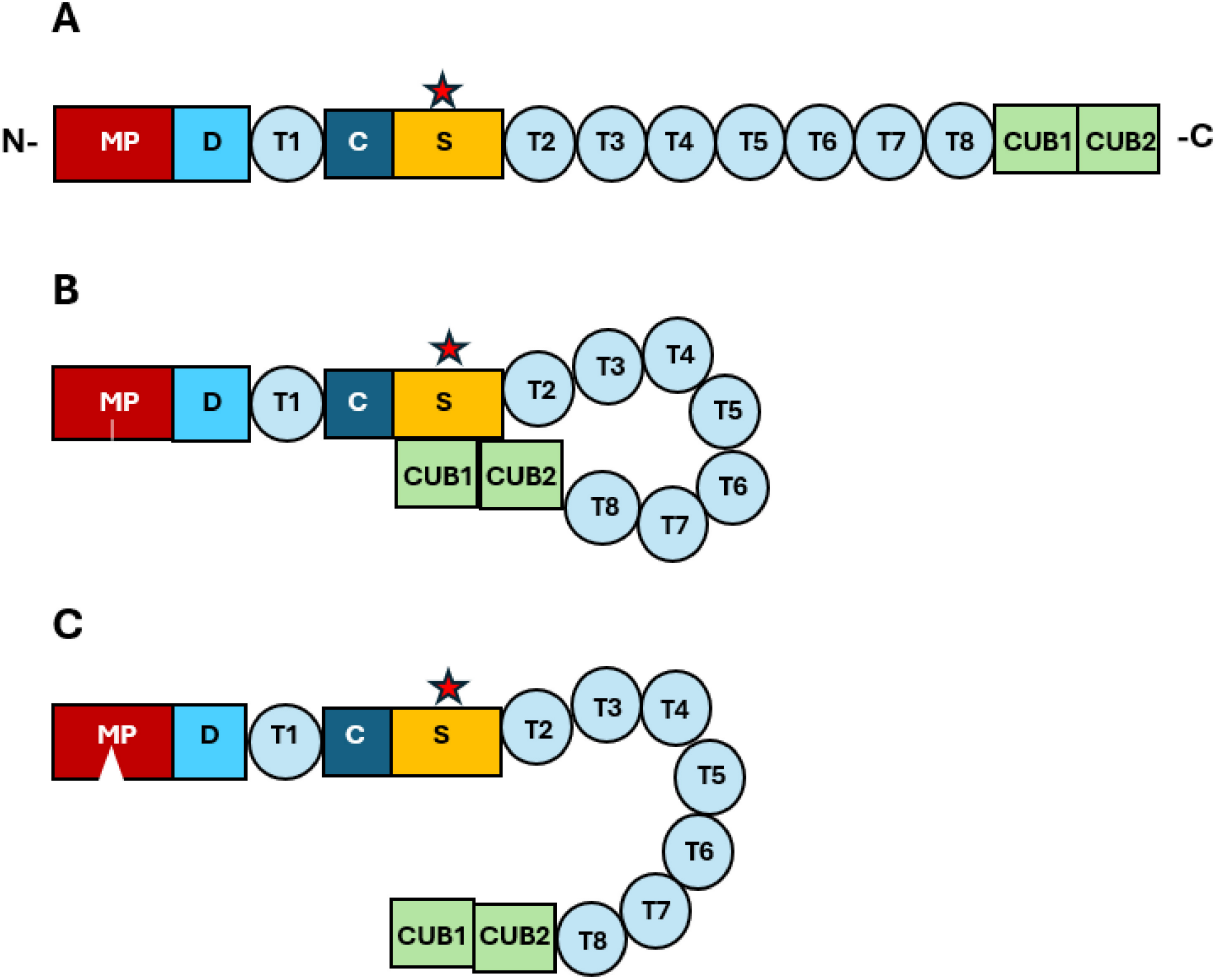
The structure of ADAMTS13. **(A)** ADAMTS13 is composed of 14 domains. From N terminus to C terminus: metalloprotease domain (MP, crimson rectangle), disintegrin domain (D, blue square), cysteine-rich domain (C, navy square), thrombospondin repeats (T1-8, light blue circle), spacer domain (S, yellow rectangle), and CUB (complement C1r/C1s, Uegf, Bmp1) domains (CUB1 and CUB2, green rectangles). The antibodies against ADAMTS13 in iTTP patients may target any domain, but spacer is the most frequently affected (shown as red star). **(B)** Latent conformation of ADAMTS13 in circulation. The global latency is hypothesized to be sustained by interaction of the CUB domains with spacer domain. **(C)** Activated ADAMTS13 with open and activated conformation. Under shear force, CUB dissociates from the spacer region, hypothetically increasing the probability of VWF binding; similarly, conformational changes induced by ligand binding appear to allosterically affect the catalytic site (shown as opening in M domain).

The metalloprotease contains 1,427 amino acid residues and is composed of 14 domains (metalloprotease, disintegrin, thrombospondin 1, cysteine-rich, spacer, thrombospondin 2–8, CUB1 and CUB2) (Figure 1) (8, 42). ADAMTS13 is a highly dynamic protein with multiple intrinsically disordered regions (43). It is currently hypothesized that it circulates in a latent state with the CUB domains closely apposed to the spacer-Cys-rich region until undergoing conformational changes in conditions of high shear, such as the vessel narrowing that occurs during the development of VWF-rich thrombi (44–48). Recent advances by our group and others identified conformation-altering allosteric effects of antibody binding to ADAMTS13 as central to the pathophysiology of the disease (47, 49–53).

ADAMTS13 activity is the most crucial biomarker for the confirmation of TTP diagnosis. The assays directly reflect the ability of VWF multimer cleavage at Y1605-M1606 by the metalloprotease in plasma. When plasma ADAMTS13 activity is severely deficient (< 10 IU/dL, or < 10% of normal) with or without detectable anti-ADAMTS13 IgG or inhibitors, in the proper clinical context, the diagnosis of TTP is confirmed (20). It is also essential for assessment during follow up in remission and prognosis prediction. As an example, iTTP patients with severe deficiency of ADAMTS13 activity combined with high inhibitor titers are at a significant higher risk of exacerbation or recurrence during remission (31, 32, 54–57). ISTH guidelines recommended administration of rituximab to iTTP patients with persistently low ADAMTS-13 activity (< 10 IU/dL or 10% of normal) to prevention of relapse (27). Accordingly, due to the importance of ADAMTS13 activity measurement, the development of different assays has been explored over more than 20 years.

### 2.1 Pre-FRETS era

All ADAMTS13 activity assays are based on VWF cleavage by ADAMTS13 (20, 58–61). VWF employed in assays in early years was full-length plasma derived from VWF or synthesized VWF peptide. Options included collagen binding assays (CBA), immunoblotting assays, ristocetin cofactor activity assays, and immune radiometric assays (20, 58, 59). Some of these tests showed high sensitivity and specificity, e.g., the specificity of and sensitivity of CBA being 100 and 93%, respectively (20, 62, 63). However, such assays were technically challenging and time-consuming, required skilled laboratory specialists, and often required the utilization of strong denaturants which potentially distorted activity results, hindering their widespread application in clinical settings (58, 59, 64).

### 2.2 Modern ADAMTS13 activity assays

**FIGURE 2:**
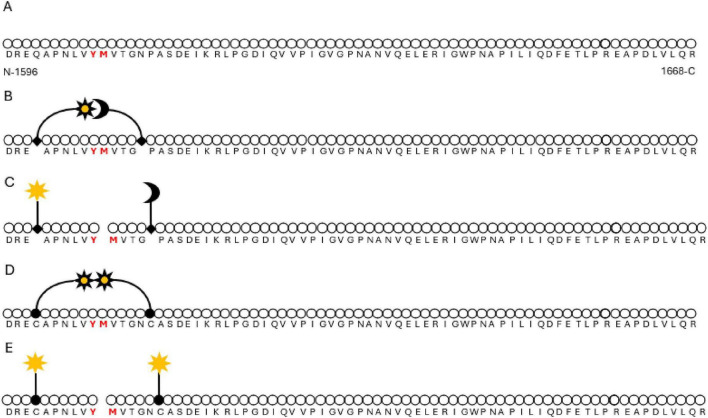
FRET (fluorescence resonance energy transfer) assay of VWF73. **(A)** Structure of VWF73. VWF73 is a short peptide from the A2 domain of VWF, composed of 73 amino acid residues, from D1596 at the N terminus to R1668 at the C terminus, including the cleavage site Y1605-M1606. **(B,C)** FRET assay principles. In the original design by Kokame et al., Q1599 and N1610 were substituted by 2,3-diaminopropionic residue (A2pr, shown as solid diamonds), and modified by 2-(Nmethylamino) benzoyl group (Nma, shown as star) and 2,4- dinitrophenyl group (Dnp, shown as moon), separately. Fluorescence resonance energy transferring excited Nma is quenched by Dnp **(B)**. When the Y1605-M1606 scissile bond is cleaved by ADAMTS13, the quencher and acceptor are separated, and the fluorescence emitted from Nma is detected **(C)**. **(D,E)** In our modified design, Q1559 and P1611 are substituted by cysteines (shown as solid circles) and modified by fluorescein-5-maleimide (shown as stars), which auto-quench when the peptide is intact **(D)**. When VWF73-Cy2 is cleaved, auto-quench is abolished, resulting in fluorescence emission **(E)**.

In 2004, Kokame et al. reported a 73 amino acid peptide consisting of the residues from D1596 to R1668 in the VWF A2 domain could be utilized as a specific substrate for ADAMTS13 cleavage (65). The following year saw the seminal introduction of a fluorescence resonance energy transfer (FRETS) assay for ADAMTS13 activity using a fluorescently labeled version of the aforementioned peptide (VWF73) (Figure 2) (60). The assay proved to be sensitive to ADAMTS13 activity, with a lower limit of detection of ADAMTS13 activity < 3–5%, sufficient to detect severe ADAMTS13 deficiency (58, 59, 64, 66). ADAMTS13 activity assessed by FRET-VWF73 was consistent with other assays using full-length VWF (58, 59, 64). Most importantly, this new assay detected more patients with severe low plasma ADAMTS13 activity, which was considered to be due—at least in part—to the denaturing reagents used in other assays causing dissociation of ADAMTS13 from immune complexes and hence leading to false negative results (58, 64). ADAMTS13 activity tested by FRETS-VWF73 under flow conditions were also consistent with a low enzyme activity, which reflected more physiological circumstances (64). The excellent accuracy and practicability of this validated FRETS-VWF73 assay led to its use as the standardized measurement of ADAMTS13 activity soon after it was developed (66, 67). However, the results of FRETS-VVF73 assays could also be influenced by hyperbilirubinemia, hemoglobin, neutrophil peptides, and other conditions (66, 68–71).

**FIGURE 3:**
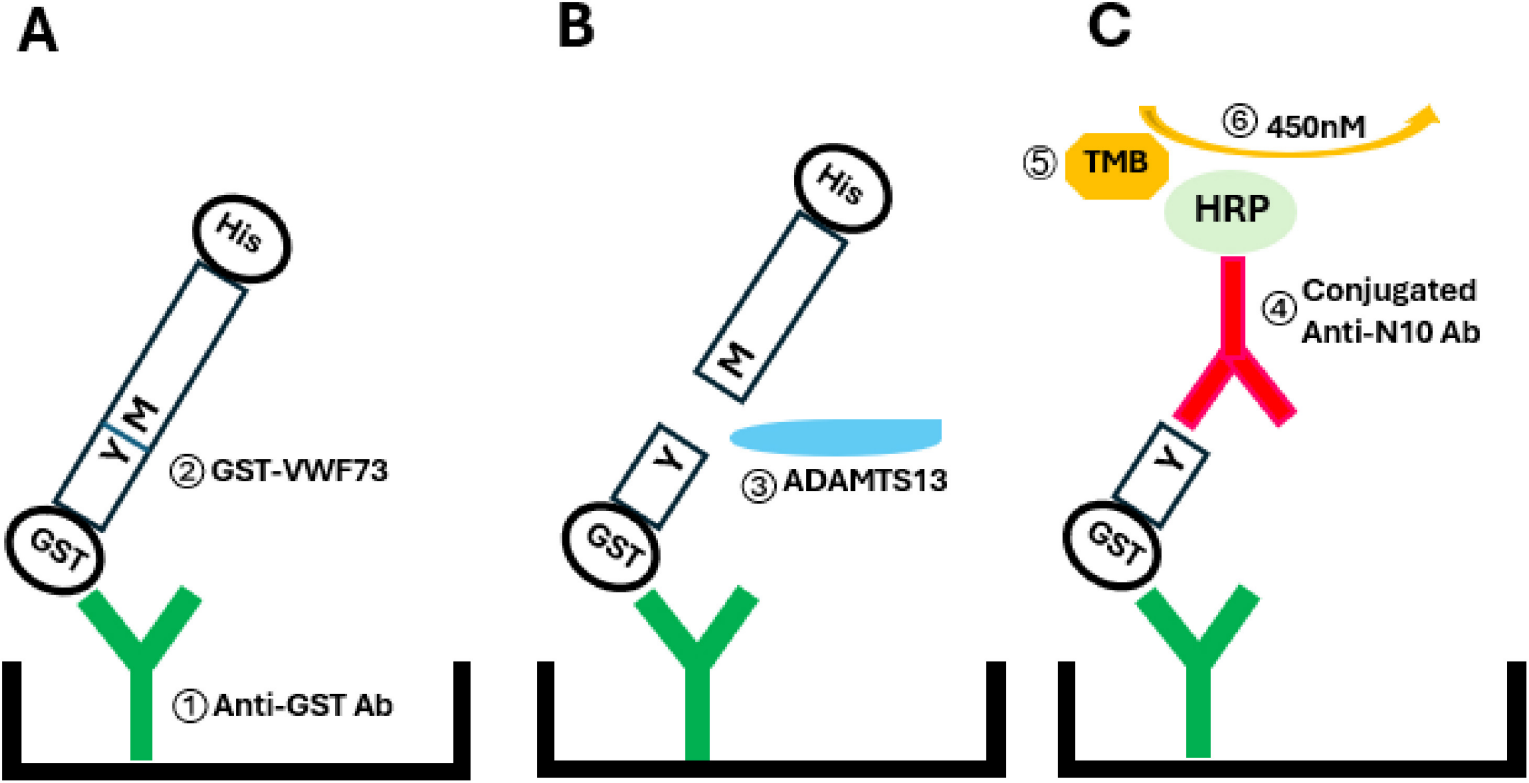
Principle of enzyme linked immunosorbent assay (ELISA) for ADAMTS13. Short peptide VWF73 is labeled by glutathione S-transferase (GSF) at N terminus and histidine (His) at C terminus. Scissile bond is at Y1605-M1606; and N10 is the labeled decapeptide from 1596D-Y1605 (10 residues of N terminus from scissile bond). **(A)** Preparation of substrate for ADAMTS13 activity ELISA. ① Anti GST antibodies are coated on the surface of microplate wells. ② GST-VWF73 is incubated in the wells. **(B)** Incubation of sample with GST-VWF73. ③ ADAMTS13 in sample cleaves substrate GST-VWF73 at Y1505-M1606. **(C)** Detection of ADAMTS13 activity. ④ Horseradish peroxidase (HRP) conjugated anti-N10 IgG antibody binds to N10 decapeptide. ⑤ HRP substrate tetramethylbenzidene (TMB) reacts with the conjugated part of antibody, causing color change in reaction system. ⑥ Reaction is stopped and microtiter plate is read at 450 nM.

Another chromogenic ADAMTS13 activity assay is an enzyme linked immunosorbent assay (ELISA) method using a N-10 monoclonal antibody to recognize decapeptide labeled VWF73 (GST-VWF73-His) cleavage production ending with the C-terminal edge residue before the scissile bond (Figure 3) (72). This assay is also currently in wide use and is less influenced by the presence of bilirubin. Based on VWF73, other VWF peptides with different lengths or amino acid residue substitutions for fluorescence labeling have been developed for laboratory research, e.g., VWF96 (44), VWF115 (73), and VWF71 (66). A surrogate substrate derived from cattle—FRETS-rVWF71—appears to be a sort of “universal” substrate susceptible to cleavage by ADAMTS13 from a wide variety of animal species, and compares favorably to commercial FRETS-VWF73 when testing the ADAMTS13 activity of human plasma (61). Our laboratory employs a facilized version of FRETS substrate. Two amino acid residues flanking the tyrosine–methionine (Y1605–M1606) ADAMTS13 cleavage site on recombinant VWF73 peptide are substituted by cysteines for fluorescein-5-maleimide labeling (Figure 2) (74). The modified VWF73-2Cys is expressed and purified in an *E. coli-*based system and behaves sensitively and specifically in FRETS assay. The convenience, economy and reproducibility of this system facilitate the study of ADAMTS13 enzyme kinetics and thermodynamics in terms of interaction between ADAMTS13 and its substrate or antibodies (47, 49, 50).

Though deficiency of ADAMTS13 activity could be caused by many disorders other than TTP, the criterion of ADAMTS13 activity < 10% has a 100% specificity for TTP, with a sensitivity > 90%, so the cut-off value is used across assays (58, 60, 64, 75–77). According to ISTH guidelines, for patients with a high index of suspicion of having TTP, when plasma ADAMTS13 activity assays < 10 IU/dL or < 10% of normal range in the proper clinical context, the diagnosis of TTP is essentially confirmed (20). ISTH TTP guidelines define the 10—20% activity range as an equivocal result and > 20% as negative for severe deficiency; and if a MAHA patient has ADAMTS13 activity > 20%, the diagnosis of HUS should be considered while other causes have been excluded (27).

### 2.3 Time of rapid assays

Since turnround time affects clinical decisions and may increase the potential risk of unwanted exposure to plasma and TPE complications for non-TTP patients (78), the ISTH states obtaining results within 72 h is ideal and 3–7 days is acceptable (20, 27). For the in-house FRETS assay, skilled technicians are still needed, and turn-around time (TAT) is still usually over one day. In the last decade, commercially available rapid assays that reduce the TAT to < 1 h have been developed, including a chromogenic enzyme-linked immunosorbent assay (ELISA) assay (Technozym, Technoclone, Vienna, Austria) and a fully automated ADAMTS13 activity chemiluminescence immunoassay (CLIA) (HemosiL Acustar, Instrumentation Laboratory, Bedford, MA, United States). Results from these rapid assays were in overall good agreement with classical in-house FRETS assay, and correlated with each other and with a low risk of bias (67, 79–87). In a systemic review and meta-analysis, the CLIA assay has a high enough sensitivity and specificity to avert empiric therapy of TTP among non-TTP patients (79).

However, it has also been reported that the CLIA assay can sometimes underestimate or overestimate ADAMTS13 activity, which might affect clinicians’ judgment (80, 82, 86), and minor discrepancies between the ELISA rapid assay and traditional FRETS-VWF73 activity assays have also been reported (67). For example, among patients’ samples with measurable activity levels, the mean bias of Acustar HemosIL assay to FRET-VWF73 assay was 12.3% (95% CI: 7.3–17.3%), reflecting possible overestimation by the HemosIL assay in some patients (86). In another study, absolute mean bias of the Technozym ELISA assay to the Acustar HemosIL assay on a Bland–Altman plot was 4.8%, although the 95% confidence interval crossed zero (95% CI –0.27–9.83%) (81). In the cohort reported by Stratmann et al., despite overall good agreement (Pearson R = 0.93, *p* < 0.001) compared to the Technozym ELISA assay, the Acustar HemosIL assay showed a trend for lower estimation of ADAMTS13 activity in the intermediate-low activity range (ADAMTS13 activity 0–10%, median 7.2% (2.9–9.8) and 4.0% (1.5–7.0), respectively, *p* = 0.001; and 10–30%, median 22.0% (10.3–29.0) and 21.2% (7.2–26.5), respectively, *p* = 0.005), and this correlation was lost when ADAMTS13 activity was above 30% (R = 0.21, *p* = 0.57) (82). Other data supports the notion that ADAMTS13 activities assessed by the Acustar HemosIL assay were significantly lower than those obtained using the Technozym ELISA assay (*p* < 0.001) and FRETS-VWF73 assay (*p* < 0.001), and although the sensitivity reported by this group was 100%, the false positive rate was 26% (5/16) in non-TTP TMA samples (80). A trend toward lower value in previously frozen plasma specimens was also observed in intra-method reliability assessment comparing fresh to frozen samples (*n* = 6) by Acustar [97.9 (77.1–120.1) and 85.7 (69.3–113.3), respectively, *p* = 0.08) (82). Although the fast and accurate values realized the wide application of these methods in clinical practice, when ADAMTS13 activity results are equivocal or discordant with the clinical information, a reference method in a specialized laboratory is still recommended (67, 81).

## 3 Genetic testing

The hereditary form of TTP is also known as Upshaw–Schulman syndrome (88). More than 200 pathogenic mutations in the ADAMTS13 gene have been identified (88, 89). Congenital TTP is usually diagnosed in children or teens, and amongst adults often during pregnancy; cTTP might account for as many as 25–50% of TTP cases in children and pregnant women (90). In the French TMA registry, during the years of 2000–2020, 29 out of 108 (27%) pregnancy-onset TTP cases were cTTP (91). In another Japanese cohort, 38% of female cTTP patients had initial diagnosis during pregnancy (92).

Widely different therapeutic strategies are utilized between iTTP and cTTP. In the former, TPE and immunosuppression are cornerstones while for the latter, patients receive ADAMTS13 supplementation via plasma infusion or recombinant human ADAMTS13 (27). An important clue to the diagnosis of hereditary TTP is the persistence of severe ADAMTS13 deficiency while patients are in clinical remission without evidence of MAHA or thrombocytopenia (88). It is recommended that for TTP patients with long-term negative anti-ADAMTS13 antibodies or inhibitor but low ADAMTS13 activity, or for patient populations where cTTP is epidemiologically more prevalent, ADAMTS13 gene sequencing should be sent to rule out the hereditary form of the disease (29). Furthermore, once the proband patient is confirmed, the family should be screened for individuals at potential risk. The database of ADAMTS13 mutations is accessible and updated in the Leiden Open Variation Database (LOVD) (93).

## 4 ADAMTS13 antigen

**FIGURE 4:**
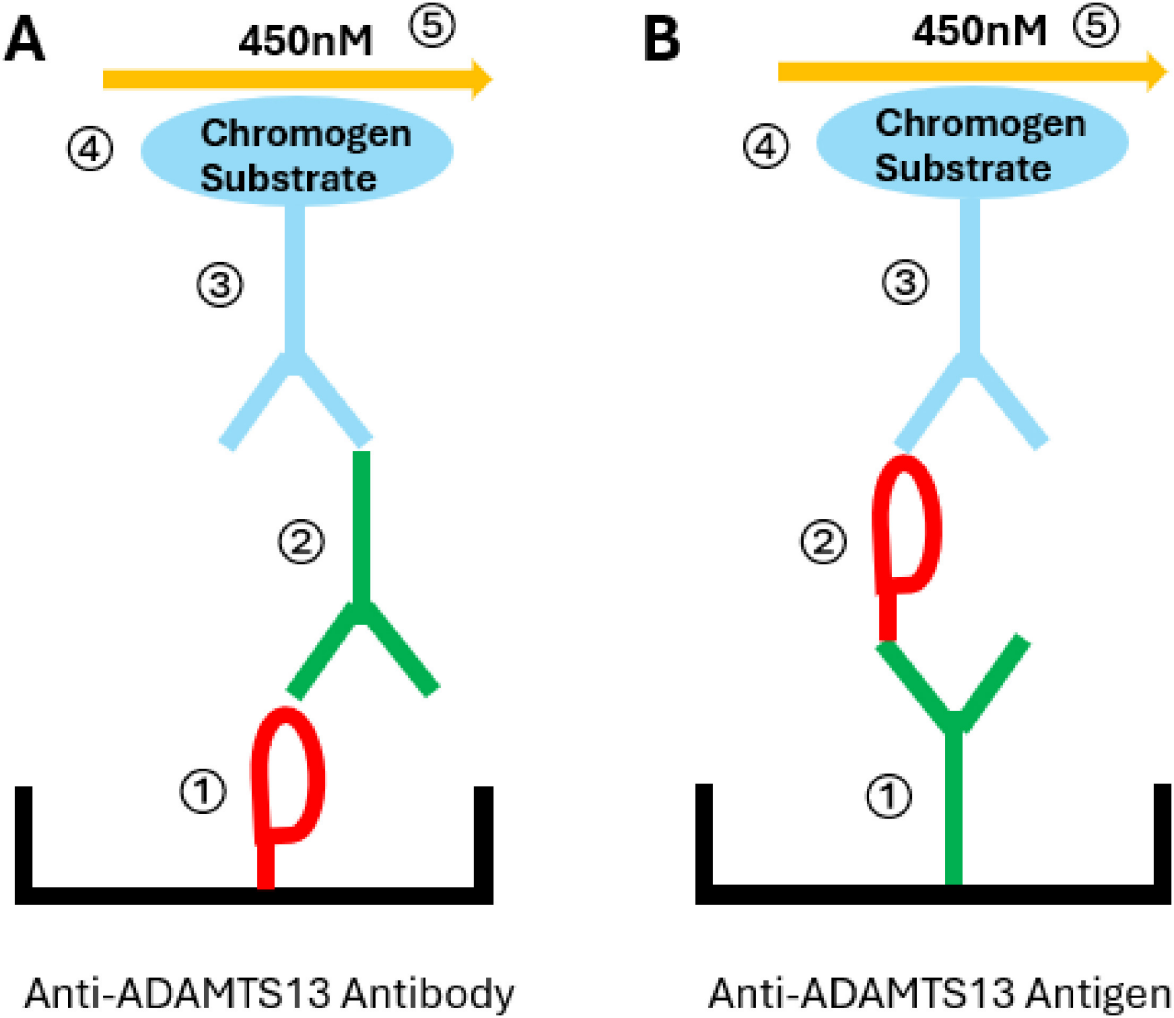
Principle of enzyme linked immunosorbent assay (ELISA) for ADAMTS13 antibody and ADAMTS13 antigen detection. **(A)** ADAMTS13 antibody ELISA. ① Recombinant ADAMTS13 molecules are coated on the surface of microplate wells. ② Plasma sample is incubated in the wells; anti-ADAMTS13 antibody binds ADAMTS13. ③ Conjugated (e.g., by streptavidin-peroxidase) anti-human IgG antibody binds to anti-ADAMTS13 antibody. ④ Chromogen substrate reacts with the conjugated part of antibody, causing color change in the reaction system. ⑤ Reaction is stopped and microtiter plate is read at 450 nM. **(B)** ADAMTS13 antigen ELISA. The wells are coated by polyclonal anti-ADAMTS13 antibodies ① to catch ADAMTS13 molecule in plasma ②, which is bound by conjugated anti-ADAMTS13 antibody ③, reacted with chromogen ④, and read at 450 nM ⑤.

ADAMTS13 antigen reflects the level of circulating enzyme in plasma. Though commercial kits or in-house methods of ADAMTS13 antigen ELISA assays are available (Figure 4), this testing is not routinely utilized for TTP diagnosis or management. However, ADAMTS13 antigen testing informs current TTP research. In iTTP, though, the role of antigen testing to quantify the role of antibody-mediated clearance in the pathophysiology of the disease remains somewhat controversial, as some reports have suggested that different techniques vary widely in their quantification of ADAMTS13 antigen (94). Such discrepant results have been speculated to be due to antibody-bound ADAMTS13 interfering with ELISA-based quantification, either because of steric interference or allosteric alteration of ADAMTS13 epitopes; stringent conditions needed to perform assays, particularly in the case of capillary Western blotting; or heterogeneity between patients in terms of the extent of the role that ADAMTS13 antigen clearance plays in their specific form of iTTP. Others have reported that anti-ADAMTS13 antibodies do not hamper ELISA-based ADAMTS13 antigenic quantification when purified anti-ADAMTS13 IgG is added to plasma, though the group does not report a mitigation strategy to exclude already antibody-bound ADAMTS13 affecting the results (95). Nonetheless, it is an important and evolving field of research, and we review recent findings and ongoing investigations in this section.

ADAMTS13 antigen correlates well with ADAMTS13 activity, particularly in patients with cTTP and those without TTP but with other reasons for lower-than-normal ADAMTS13 activity (96, 97). However, likely for reasons as speculated above, results vary in iTTP patients (96, 98). Some groups have speculated that the primary pathogenic process in iTTP is clearance of ADAMTS13 antigen, though notably only one antigen detecting technique was utilized and it is not clear if the results truly represent the concentration of circulating ADAMTS13 antigen (99). ADAMTS13 antigen combined with other biomarkers has also been investigated for evaluation of prognosis and the risk of relapse in iTTP patients. Low level of ADAMTS13 antigen predicts higher risks of exacerbation and relapses during remission or mortality, though again this may reflect an abundance of antibody-bound ADAMTS13 unable to be quantified accurately as an explanation of such results (54, 55, 57, 96, 99). Sui et al reported that low plasma ADAMTS13 antigen within 25th percentile at clinical response/remission exhibited a significantly lower exacerbation-free survival rate than those with plasma ADAMTS13 antigen in the other 3 quartiles [Hazard Ratio 3.3; 95% Confidence Intervals: 1.3–8.2; *p* = 0.01] (57). From the United Kingdom TTP registry, a prospective study demonstrated that patients with presenting antigen level in the lowest quartile (antigen < 1.5%) had a mortality of 18% compared with 3.8% for the highest quartile (antigen > 11%) (100). Thomas et al. also reported that ADAMTS13 antigen in the lowest quartile at first presentation was associated with increased mortality [OR 5.7; 95% CI 1.5–21.8; *p* = 0.01] (99). In another report, for iTTP patients who achieved initial clinical responses, ADAMTS13 antigen levels appeared to be restored faster than ADAMTS13 activity, and higher ADAMTS13 antigen levels correlated with sustained remission (96).

## 5 Evaluation of anti-ADAMTS13 antibodies

As previously mentioned, iTTP is the major form of TTP and comprises more than 95% of all TTP cases (5, 101). The confirmation of inhibitor or anti-ADAMTS13 antibody is essential for diagnosis of iTTP, influencing clinical decision making primarily regarding continuation of TPE and initiation of immunosuppression (20, 26). Though other antibody classes have been identified that can target and inhibit ADAMTS13, the vast majority of anti-ADAMTS13 antibodies in iTTP patients are of the IgG class (102, 103). The disease is polyclonal (104, 105). The ISTH recommends diagnostic testing centered on the measurement of plasma ADAMTS13 activity and the identification or quantification of inhibitor or anti-ADAMTS13 IgG in all patients with suspected or confirmed TTP (20, 27). Anti-ADAMTS13 antibody tests include the Bethesda assay, which is used to detect a functional inhibitor of ADAMTS13 but does not quantify anti-ADAMTS13 antibody antigenic levels; anti-ADAMTS13 IgG ELISA, which quantifies antibody levels without directly testing their inhibitory capacity; and immune complex detection. In another cohort, the presence during remission of both severe ADAMTS13 deficiency and anti-ADAMTS13 antibodies increased the likelihood of recurrence 3.6 times (54). Here, we review the different techniques available, both clinically and in current research, to identify and detect anti-ADAMTS13 antibodies and their inhibitory effects on the enzyme.

### 5.1 Bethesda assay

The Bethesda assay to detect an ADAMTS13 inhibitor is similar to the anti-coagulation factor testing doe for hemophilia patients with repeated exposure to exogenous coagulation factors (106). Inhibition is confirmed using serial dilutions with pooled normal human plasma (NHP) and activity is measured by a FRETS-VWF73-based activity technique; inhibitor titer is calculated based on the number of dilutions necessary to achieve normalization of ADAMTS13 activity (106). Among patients with ADAMTS13 activity < 10%, an inhibitor titer of 2 or more Bethesda units/mL has been reported to be associated with lower survival; relapse rate was greater than 34% among survivors with ADAMTS13 activity < 10% compared to 4% of those with ADAMTS13 activity ≥ 10% within 7.5 years (31). The Bethesda assay might be affected by temperature and other conditions, but the main limitation is that this method can only detect inhibitory antibodies of iTTP patients; it was reported that non-inhibitory IgG autoantibodies were detected in 29% of idiopathic and 50% of non-idiopathic TTP patients (98), and therefore a negative Bethesda assay is not able to fully exclude the diagnosis of iTTP (106, 107). Nonetheless, it is clear that the majority of patients with iTTP have inhibitors capable of interfering with ADAMTS13 activity (85, 93, 104, 108–110), which strongly suggests a major role of anti-ADAMTS13 antibody-mediated inhibition of VWF cleavage as a central pathophysiologic feature in most, if not all, iTTP patients.

### 5.2 Anti-ADAMTS13 IgG assay

Anti-ADAMTS13 IgG ELISA assays detect anti-ADAMTS13 IgG in plasma quantitatively and qualitatively, including neutralizing and non-neutralizing antibodies (Figure 4) (93). In-house and commercial testing are both employed for detection; the latter includes kits available from such companies as Technozym and HemosIL AcuStar (80, 83–85). Groups have compared the performance of these commercial options in the diagnosis of iTTP. The Technozym assay has been shown to yield a high anti-ADAMTS13 IgG titer in TTP patients and showed good agreement with reference, but discrepant results have been noted in iTTP patients in remission with ADAMTS13 activity 10–50% (84, 85). Though this kind of discrepancy does not currently affect management of patients already diagnosed with iTTP, as immunosuppression is already utilized in such scenarios, it highlights the need for novel or improved techniques to detect antibody activity before ADAMTS13 activity is affected. When compared to one another, the two commercially available Technozym ELISA and Acustar HemosIL assays had good comparability by linear regression analysis (*r* = 0.987; *P* < 0.001) with a slight positive bias (average bias = 1.703) using the HemosIL assay (83).

Though clearly capable of distinguishing iTTP from cTTP, quantification of anti-ADAMTS13 IgG has shown discrepant results in terms of its quantitative use as a prognostic or predictive marker for iTTP patients. In some reports, anti-ADAMTS13 antibody titers positively correlated with increased relapse rate or mortality during remission, always accompanied by severe ADAMTS13 activity or low levels of ADAMTS13 antigen (24, 31, 32, 54, 55). In the UK registry, patients with ADAMTS13 antibody levels in the upper quartile had a mortality of 16.9% compared with 5.0% for the lowest quartile; when the parameters were combined, patients with a low ADAMTS13 antigen and high anti-ADAMTS13 antibody levels had a reported mortality of 27.3% compared with 5.6% of those with high ADAMTS13 antigen and low anti-ADAMTS13 antibody (24). In the cohort reported by Sui et al., high anti-ADAMTS13 IgG > 75th percentile was also predictive of exacerbation recurrence (57). However, in other cohorts, quantification of anti-ADAMTS13 antibody or inhibitor had no impact on clinical outcomes in iTTP patients (5, 56). Again, these reports emphasize the need for further research into the potential role of antibody quantification in the management of iTTP patients.

It is important to note that negative anti-ADAMTS13 IgG testing does not fully rule out iTTP, for antibodies may bind to ADAMTS13 as immune complexes, accelerating their clearance from circulation (111). Patients’ anti-ADAMTS13 IgG may subsequently become detectable during remission or relapse (112). It is also notable that non-specific anti-ADAMTS13 antibodies may exist in about 4% healthy individuals, and 5–13% in thrombocytopenia from other causes and autoimmune diseases (103). We hope it is clear to the reader that, despite the massive advances made in the field in the last several decades, much work yet remains to understand iTTP.

### 5.3 ADAMTS13-specific immune complexes

Immune complexes (IC) have been identified in iTTP patient plasma for more than 40 years. Anti-ADAMTS13 antibodies bind to their target epitopes and form ADAMTS13-specific IC, accelerating the clearance of ADAMTS13 from circulation (111, 113–116). However, ADAMTS13-specific IC could be cleared by TPE (116). Like ADAMTS13 antigen, ADAMTS13-specific IC is not routinely tested at presentation, but their presence may interfere with the detection of anti-ADAMTS13 IgG and yield false negative results, thus affecting clinical management (112). No commercial kit is available to detect ADAMTS13-specific IC. All studies on ADAMTS13-specific IC have been performed using ELISA-based assays developed by different laboratories (111, 113, 114, 117). In some reports, the prevalence of IC in iTTP patients could be as high as 97% in the acute phase (113), while other groups report quantifiable ADAMTS13-specific IC prevalence of 39–43% (111, 114). In some studies, ADAMTS13-specific IC was negatively associated with anti-ADAMTS13 IgG titer as well as the inhibitor titer in plasma (114, 117), and positively associated with ADAMTS13 antigen (114), yet similar correlations were not found from another cohort in the same patient registry (111). In these studies, ADAMTS13-specific IC was not related to severity of disease at presentation, but patients with elevated level of ADAMTS13-specific IC had higher risk of relapse within 2 years of their first iTTP episode and needed more TPE sessions to attain remission (111, 114). Per Ferrari et al., IgG4 was the most prevalent subclass in ADAMTS13-specific IC, and persisted during remission for a period of years; over that time, the ADAMTS13-specific IC level of all other subclasses (IgG1-3) decreased and eventually became undetectable (113).

### 5.4 IgA and IgM anti-ADAMTS13 antibodies

The predominant isotope of anti-ADAMTS13 antibody is IgG (32, 55, 98, 102, 118, 119), but anti-ADAMTS13 IgA and IgM have also been reported (32, 55, 103, 119, 120). The presence of non-IgG anti-ADAMTS13 antibodies presents a significant diagnostic challenge in iTTP patients, particularly for patients with negative Bethesda assays and a high suspicion for iTTP rather than cTTP. In 2003, non-neutralizing anti-ADAMTS13 IgM was found in a patient with TMA and severely low ADAMTS13 activity, consistent with a diagnosis of TTP; the presence of the anti-ADAMTS13 IgM was presumed to confirm iTTP in this patient (120). Analyzing a cohort of 59 TMA patients, Rieger et al., found 35 patients had positive anti-ADAMTS13 IgG in the acute phase, while 4 patients had anti-ADAMTS13 IgM (103). Some reports suggest anti-ADAMTS13 IgA might be related to the severity of disease. For example, Bettoni et al. reported that higher anti-ADAMTS13 IgA, IgG1 and IgG3 titers were associated with lower platelet counts (55), and in a French cohort of 35 iTTP patients who experienced TMA with ADAMTS13 activity < 50%, 3 of the patients had high titers of anti-ADAMTS13 IgA; each were reported to have died during the TMA episode (32). Pos et al. reported that in 48 TTP patients with a high titer of anti-ADAMTS13 antibodies, 5 patients had detectable anti-ADAMTS13 IgM, and 9 patients had detectable anti-ADAMTS13 IgA; all anti-ADAMTS13 IgA were of the IgA1 subclass (119). Though it is a rare scenario, when anti-ADAMTS13 antibody is negative, the existence of other isotypes of IgM and IgA also should be considered when there is a moderate to high index of suspicion of an immune component of TTP; however, in most cases, anti-ADAMTS13 IgG antibodies also appear to be present in patients with positivity of other antibody isotypes (21).

### 5.5 IgG subclasses of anti-ADAMTS13 specific antibodies

As mentioned above, IgG is the most predominant isotype of anti-ADAMTS13 specific antibody in iTTP; though all 4 subclasses (IgG1-4) of anti-ADAMTS13 IgG exist in iTTP patients plasma, IgG1 and IgG4 are by far the most prevalent (55, 113, 118, 119, 121–123). In some studies, anti-ADAMTS13 specific IgG4 has been reported to be the most prevalent subclass in iTTP and is detectable in over 90% of patients, persisting during remission (113, 118), while others report that detectable anti-ADAMTS13 specific IgG4 and IgG1 levels among iTTP patients are similar (119, 121, 122). Most patients produce anti-ADAMTS13 IgG1 antibodies in the acute phase of iTTP, while in some patients both IgG1 and IgG4 predominate, and yet in others, only IgG4 anti-ADAMTS13 antibodies exist (113, 118, 119). Clinical studies have associated IgG1 with acute episode severity, while IgG4 was once considered “protective” (118). However, other studies found that IgG4 levels correlated with inhibitory potential (124).

Although anti-ADAMTS13 IgG subclass predominance appears to vary between patients (55, 113, 118, 119, 121–123), further research is needed to determine the pathophysiologic significance of this variability. Compared with IgG1, IgG4 has a less flexible Fc region in part due to non-canonical disulfide bonds (125). Unlike IgG1, IgG4 does not activate complement and interacts weakly with effector cells (126, 127). Interestingly, IgG4 undergoes Fab arm exchange, and the vast majority of IgG4 essentially circulates as monovalent ligand (127). The implications of these subclass differences are areas of active research. In previous work, we demonstrated the effects of antibody variable loop binding upon ADAMTS13 activity using single chain fragments of the variable loop (scFv’s) developed from a phage display library derived from iTTP patients, and found that these primarily affect enzymatic turnover (47, 49). In ongoing work, we are exploring whether mechanistic effects of IgG with the same complementarity determining regions (CDR) as the scFv’s used in our previous work differ based on subclass, or if antibody valency influences ADAMTS13 activity.

**FIGURE 5:**
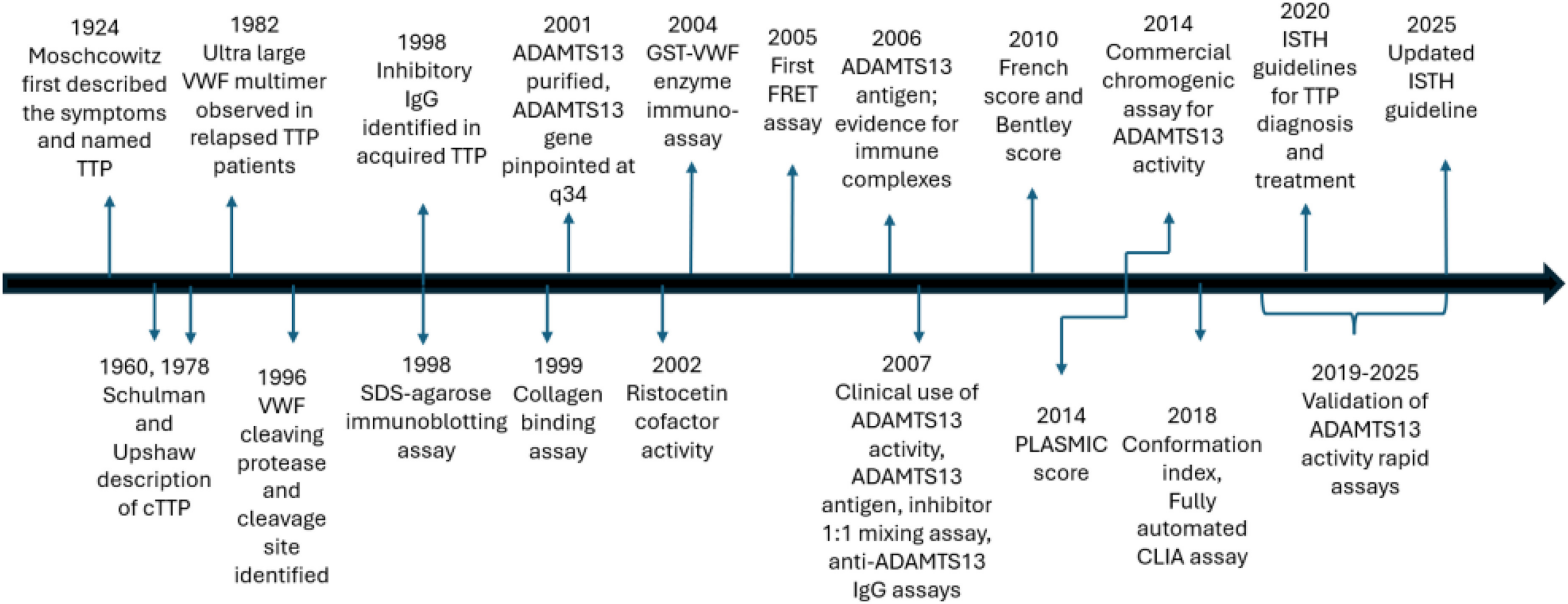
Timeline of significant events in the history of TTP and the development of ADAMTS13-based laboratory testing.

Significant advances have been made over the years regarding our understanding about the function of ADAMTS13 and its inhibition in iTTP (Figure 5). In the next section, we discuss some of the research into ADAMTS13-related testing with the potential to motivate future advances in the field.

## 6 Vanguard of research: conformational and allosteric effects on ADAMTS13 function and inhibition in iTTP

In recent years, the importance of ADAMTS13 conformation in the pathophysiology of iTTP has emerged. As a protein with multiple intrinsically disordered regions which may bind its substrate in various conformational orientations (43), ADAMTS13 has proven to be a difficult enzyme to comprehensively characterize using traditional molecular structural methods. No full-length structure of ADAMTS13 has been published to date. Nonetheless, significant progress has been made in the last decade in terms of our understanding of the local and global effects of both intra- and intermolecular interactions involving ADAMTS13 on the pathophysiology of iTTP. Allostery, in which ligand binding influences conformational effects in distal domains, plays a powerful role in both the normal function of the protein as well as its inhibition by antibodies (43, 46–50, 104, 128). Some evidence also suggests that ADAMTS13 is capable of forming functional multimers (50, 104, 129), with the implication that binding to a given monomer within an ADAMTS13 complex could affect the conformation of adjacent monomers. In this section, we review the cutting-edge research providing new insights into antibody-mediated effects on ADAMTS13 activity and its inhibition in iTTP, and how this work promises to yield new diagnostic and therapeutic approaches for patient care.

### 6.1 Physiological ADAMTS13 activity: features, insights, and caveats relevant to iTTP

To better understand recent insights into how antibodies affect ADAMTS13 in iTTP, we first review some aspects of the physiological activity of the enzyme. ADAMTS13 regulation is thought to be maintained by global and local latency (51). The global latency is hypothesized to be sustained by interaction of the CUB and spacer domains, thus making ADAMTS13 adopt a “closed” conformation in circulation by default and prevent off-target cleavage (130, 131). In the currently accepted model, this global latency is alleviated in conditions of high shear, such as the formation of a nascent platelet plug, when ADAMTS13 CUB domains bind to the D4-CK domains of VWF. This interaction essentially “opens” ADAMTS13, allowing for VWF A2 domain binding to the important spacer domain substrate binding exosite and subsequent cleavage by the metalloprotease domain (48). Local latency is thought to occur via the ionic interaction of the “gatekeeper triad” of Arg193, Asp217 and Asp252 in the metalloprotease domain, which blocks the active-site cleft (44, 132). ADAMTS13 is thought to shift to a transient “open” active site conformation via a molecular zipper mechanism when exosite substrate binding of the VWF A2 domain occurs in the proximal ADAMTS13 domains, leading to allosteric activation via “unlocking” the gate-keeper triad, allowing for VWF cleavage at the exposed scissile bond (44).

A unique feature of ADAMTS13 activity assays which we have heretofore yet to mention is that diagnostic testing is performed in conditions significantly more acidic and at a lower temperature than present in human plasma in metabolic homeostasis (∼pH 7.4). As an example, the optimized condition for the FRETS-VWF73 assay is pH 6.0 and 25–30°C, or diagnostic conditions (60). Enzymatic studies revealed that at 25°C, FRETS-VWF73 acts as a more “sticky substrate” than at a more physiologic temperature of 37°C (133). Atomic force microscopy (AFM) and hydrogen-deuterium exchange mass spectrometry (HX-MS) experiments have demonstrated that the conformation of ADAMTS13 appears to “open” in conditions more acidic than pH 7.4, suggesting that acidity level affects the interaction of the CUB and spacer domains (134, 135). Further evidence that the CUB-spacer interaction may be affected by pH comes from ADAMTS13 activity assays comparing a truncated form of ADAMTS13 containing only the five N-terminal domains—metalloprotease, disintegrin, TSP1, cys-rich, and spacer (MDTCS)—with full-length recombinant human ADAMTS13. For the MDTCS form, change in pH (range: pH 6.0–pH 7.4) did not affect relative VWF cleavage activity, but activity dramatically decreased with increasing pH for the full-length construct.

### 6.2 Antibody-mediated effects on ADAMTS13 activity

It is hypothesized that pH and temperature may play an important role in how antibodies affect ADAMTS13 in iTTP patients (47, 49, 50). Since ADAMTS13 is highly conformationally dynamic, subtle changes in the local and global environments of the protein may significantly affect antibody binding to ADAMTS13 and influence its function. As diagnostic conditions do not recapitulate the environment of plasma, we may have a somewhat distorted view of how ADAMTS13 inhibition works *in vitro* vs. *in vivo*, even though ADAMTS13 activity testing has clearly been validated as a way of diagnosing people with the disease and identifying those who would benefit from TTP-directed interventions. Teasing apart the effects of individual anti-ADAMTS13 antibody clones in a given iTTP patient is both a tantalizing goal to be able to develop specific diagnostic and therapeutic approaches that can be tailored to individuals, and also a massive challenge.

Recent work has provided new insights into how antibodies may affect ADAMTS13 differently in different assay conditions. In our aforementioned studies using scFv’s derived from iTTP patients (105), we have tested ADAMTS13 activity in different conditions of acidity and temperature (47, 49, 50). By using FRETS-VWF73 activity assays and Michaelis-Menten-based analytical approaches, we found that regardless of reaction conditions (pH 6 or 7, and temperature 25 or 37°C), antibodies primarily affected catalytic turnover, or *k*_*cat*_, rather than the substrate concentration at which enzyme activity is half maximal, or *K_0_._5_*, which we use as a more precise term than *K*_*M*_ in our quantitative approach to explore the thermodynamics of the system (136). We utilized HX-MS to show that spacer domain-binding antibody inhibitors effect allosteric conformational changes in the metalloprotease domain (49). Ongoing work is exploring if, whether, and to what extent pH and temperature influence the mechanistic effects of full-length IgGs, as well as any role that antibody subclass plays in variable reaction conditions.

In our previous work, we also identified scFv’s that target the C-terminal domains as stimulatory ligands. As iTTP is a polyclonal disease, we tested the effects of combining both stimulatory and inhibitory ligands, and found that in this scenario, inhibition predominates; in this same work, we were able to show one stimulatory scFv, which we dub scFv4-41, binds to the CUB domains using HX-MS (47). Nonetheless, the existence of antibodies isolated from iTTP patients that actually increase the efficiency of ADAMTS13-mediated VWF cleavage begs the question as to whether they are having some other effect on ADAMTS13 to inhibit other aspects of its function—possibly by interfering with the D4-CK interaction, for example—or if they develop as a compensatory response when inhibitors are present. Considering yet another hypothesis, we have found strong positive cooperativity when titrating inhibitor in the presence of a fixed concentration of stimulatory scFv, suggesting that ADAMTS13 may form a complex like hemoglobin with monomer-monomer interactions influencing global enzymatic effects (50, 104). We are also exploring the functional stoichiometry of ADAMTS13 and the influence of antibodies, if any, on stoichiometry and/or CUB domain interactions with VWF in our ongoing studies.

A group in Belgium has developed a series of murine anti-human anti-ADAMTS13 antibodies that target each domain of ADAMTS13, and found that several antibodies that target not only the C-terminal domains, but the spacer region as well, are capable of stimulating ADAMTS13 activity *in vitro* (21, 52, 123, 137–139). In the next section, we discuss how they were able to use these antibodies to derive new insights into the pathophysiology of iTTP, and the open questions yet to be answered in this exciting phase of exploration in the field.

### 6.3 Conformation index

Based on the above principles, the Belgian group has developed a technique to identify “open” and “closed” ADAMTS13 conformations in iTTP, and to determine how these conformations influence the pathophysiology of the disease. Using the murine anti-human anti-ADAMTS13 antibody library they developed, they identified one that recognizes an epitope on the spacer domain that binds to a truncated form of ADAMTS13 containing only the five N-terminal domains—metalloprotease, disintegrin, TSP1, cys-rich, and spacer (MDTCS)—but not full-length ADAMTS13 in normal circumstances (21, 52, 123, 137–139). This cryptic epitope is exposed in the presence of another murine anti-human antibody that binds the CUB domains, leading to an “open” conformation detectable via an ELISA assay using the cryptic epitope-binding antibody. Interestingly, this ELISA assay was found to be positive in the majority of patients with acute phase iTTP, but not for controls, patients with non-TTP TMA, or those in iTTP remission (140). A follow up study by a group in Japan appears to confirm the predominance of the “open” conformation in iTTP patients (123). Pooled anti-cys-rich/spacer domain and anti-CUB antibodies from iTTP patients capable of “opening” ADAMTS13 have also been reported by the group (52), though specific epitopes targeted by “opening” antibodies from these pooled samples have not yet been identified.

Longitudinally, a detectable “open” conformation of ADAMTS13 using the cryptic epitope-based ELISA precedes a drop of ADAMTS13 activity or antigen, even without detectable anti-ADAMTS13 IgG antibodies, before clinical relapse (138, 139). For iTTP patients with ADAMTS13 activity between 10 and 20%, when clinical decision making depends on empirical judgment, conformation index might be a potential tool for determining which of these patients need preemptive treatment to prevent clinical iTTP relapse (140).

However, using the same cryptic epitope-binding antibody and the “opening” CUB domain antibody, the group found that the “opening” murine CUB antibody also stimulates activity as well as enhancing cryptic spacer domain epitope binding (53). Similar to our findings using scFv’s derived from humans, the group reported that *k*_*cat*_ rather than substrate-related thermodynamics was primarily affected by stimulatory antibodies. This results in an apparent paradox, as “open” conformation predominates in iTTP patients, who have little to no ADAMTS13 activity, yet this same conformational state also enhances VWF cleavage by ADAMTS13 *in vitro*. Currently, it is hypothesized that both stimulatory and inhibitory antibodies likely induce global conformational changes in ADAMTS13, and that allostery plays a central role in antibody-mediated effects on VWF cleavage by the enzyme. We use “open” in quotes for this reason, as it is highly likely that ADAMTS13 can achieve multiple conformations capable of binding cryptic epitopes and that multiple ligand-induced stable conformations likely exist in any given population of ADAMTS13, whether *in vitro* or *in vivo*. As mentioned above, multiple possible explanations for the combined effects of polyclonal antibodies may prove to be correct, and we endeavor to discover the most “important” of these classes of antibodies in terms of developing tailored diagnostic and therapeutic approaches to treat iTTP.

## 7 Conclusion

ADAMTS13 is an endlessly fascinating molecule and central to the pathophysiology of TTP, both in the congenital form (cTTP) and the more common immune form (iTTP). Since the initial discovery of the disease in 1924, countless scientists and clinicians have cared for patients with this previously devastating diagnosis, and their collective collaboration has led to not only the discovery of ADAMTS13, but a wide array of laboratory techniques based on the protein that aid in the management of these patients. In this review, we endeavored to provide an overview of how these tests work, the clinical data supporting their use, and the questions they have helped us answer and will continue to help us answer in the future to improve the lives of patients with suspected or confirmed TTP.
